# Quantitative glycoproteomics analysis identifies novel FUT8 targets and signaling networks critical for breast cancer cell invasiveness

**DOI:** 10.1186/s13058-022-01513-3

**Published:** 2022-03-18

**Authors:** Cheng-Fen Tu, Fu-An Li, Ling-Hui Li, Ruey-Bing Yang

**Affiliations:** 1 Institute of Biomedical Sciences, Academia Sinica, 128 Academia Rd., Sec. 2, Taipei, 115201 Taiwan; 2 Biomedical Translation Research Center, Academia Sinica, Taipei, 115202 Taiwan; 3 Ph.D. Program in Drug Discovery and Development Industry, College of Pharmacy, Taipei Medical University, Taipei, 110301 Taiwan

**Keywords:** Breast cancer, Core fucosylation, FUT8, Glycoproteomics, Metastasis

## Abstract

**Background:**

We recently showed that fucosyltransferase 8 (FUT8)-mediated core fucosylation of transforming growth factor-β receptor enhances its signaling and promotes breast cancer invasion and metastasis. However, the complete FUT8 target glycoproteins and their downstream signaling networks critical for breast cancer progression remain largely unknown.

**Method:**

We performed quantitative glycoproteomics with two highly invasive breast cancer cell lines to unravel a comprehensive list of core-fucosylated glycoproteins by comparison to parental wild-type and FUT8-knockout counterpart cells. In addition, ingenuity pathway analysis (IPA) was performed to highlight the most enriched biological functions and signaling pathways mediated by FUT8 targets. Novel FUT8 target glycoproteins with biological interest were functionally studied and validated by using LCA (*Lens culinaris agglutinin*) blotting and LC–MS/MS (liquid chromatography–tandem mass spectrometry) analysis.

**Results:**

Loss-of-function studies demonstrated that FUT8 knockout suppressed the invasiveness of highly aggressive breast carcinoma cells. Quantitative glycoproteomics identified 140 common target glycoproteins. Ingenuity pathway analysis (IPA) of these target proteins gave a global and novel perspective on signaling networks essential for breast cancer cell migration and invasion. In addition, we showed that core fucosylation of integrin αvβ5 or IL6ST might be crucial for breast cancer cell adhesion to vitronectin or enhanced cellular signaling to interleukin 6 and oncostatin M, two cytokines implicated in the breast cancer epithelial–mesenchymal transition and metastasis.

**Conclusions:**

Our report reveals a comprehensive list of core-fucosylated target proteins and provides novel insights into signaling networks crucial for breast cancer progression. These findings will assist in deciphering the complex molecular mechanisms and developing diagnostic or therapeutic approaches targeting these signaling pathways in breast cancer metastasis.

**Supplementary Information:**

The online version contains supplementary material available at 10.1186/s13058-022-01513-3.

## Background

Breast cancer is the most common cancer and the leading cause of cancer death in women worldwide [1]. Most deaths from breast cancer are due to the metastasis of tumor cells from a primary tumor to a secondary site [2]. Glycosylation is the stepwise procedure of covalent attachment of oligosaccharide chains to proteins or lipids. The carbohydrate structures of cell-surface glycoconjugates play an important role in many physiological and pathological events, including cell growth, differentiation, and transformation [3].

Fucosylation is one of the most important types of glycosylation in cancers. Altered fucosylation has been implicated in malignant transformation, invasion, and metastasis of certain types of cancers, such as hepatocellular, gastric, pancreatic, prostate, and colorectal cancers [4–8]. To date, 13 different fucosyltransferases (FUTs) have been identified in the human genome [9]. FUT8 is the only fucosyltransferase involved in core fucosylation (addition of fucose in α-1,6-linkage to the innermost *N*-acetyl glucosamine of *N*-glycans) [10]. Our recent study showed that upregulated FUT8 during transforming growth factor (TGF)-β–induced epithelial–mesenchymal transition (EMT) represents a novel, vicious feed-forward loop linking core fucosylation and TGF-β receptor signaling to promote EMT and breast cancer progression [11]. Besides TGF-β receptors, other FUT8 targets such as epidermal growth factor receptor and an adhesion receptor complex, α3β1 integrin, may potentiate ligand binding ability and enhance downstream signal pathways to support tumor growth and metastasis [12, 13]. The pathological processes are a combination of complicated interactions of protein networks. Therefore, we need to identify additional FUT8 target proteins and their downstream signaling during breast cancer progression.

In this study, we used the comparative glycoproteomics to facilitate the identification of 392 core-fucosylated glycoproteins from two highly invasive breast cancer cell lines (MDA-MB-231 and Hs578T); 140 proteins were commonly shared targets and more than 90% were membrane glycoproteins. Ingenuity Pathway Analysis revealed that these target proteins form complex signaling networks involved in controlling downstream cellular functions including proliferation, adhesion, migration, and invasion of breast cancer cells. In addition, we verified integrin αvβ5 and interleukin 6 signal transducer (IL6ST) as novel glycoprotein targets of FUT8. Core fucosylation of these molecules played important functions in breast cancer cell adhesion to vitronectin and their responsiveness to IL-6 or oncostatin M (OSM) signaling. Further understanding the signal pathways and biological effects mediated by these core-fucosylated glycoproteins could help in deciphering the complex molecular mechanisms in breast cancer metastasis and lead to more effective diagnostic or therapeutic interventions.

## Methods

### Cell culture

MDA-MB-231, Hs578T, and HEK-293 cell lines were obtained from the American Type Culture Collection (Manassas, VA). HEK-293 cells, human breast cancer MDA-MB-231 and Hs578T cells were propagated in DMEM with 10% fetal bovine serum (FBS).

### Immunoprecipitation, western blot analysis, and lectin blot analysis

Two days after transfection, cell lysates were clarified by centrifugation at 10,000×*g* for 20 min at 4 °C. Samples were incubated with indicated antibody and 50% (v/v) Protein A-agarose (Thermo Fisher Scientific, Waltham, MA) for 2 h with gentle rocking at 4 °C. Precipitated complexes were solubilized by boiling in Laemmli sample buffer, fractionated by SDS-PAGE, and transferred onto PVDF membranes, which were blocked with phosphate buffered saline (PBS; pH 7.5) containing 0.1% gelatin and 0.05% Tween 20 and blotted with the indicated antibodies. After washes, blots were incubated with horseradish peroxidase-conjugated goat anti-mouse/rabbit IgG (Jackson ImmunoResearch Laboratories, West Grove, PA) for 1 h. Reactive bands were visualized by using the VisGlow chemiluminescent substrate, horseradish peroxidase system (Visual Protein, Taiwan). For lectin blot analysis, membranes were blocked with PBS (pH 7.5) containing 0.1% gelatin and 0.05% Tween 20, detected with biotinylated *Lens* *culinaris agglutinin * (LCA) lectin (Vector Laboratories, Burlingame, CA), then incubated with horseradish peroxidase-conjugated streptavidin (Vector Laboratories).

### Flow cytometry

Cells were collected and suspended in PBS, 2% FBS in a volume of 0.5 ml. Cell suspensions were incubated with fluorescein-labeled LCA (Vector Laboratories) on ice for 1 h. After a washing with ice-cold PBS three times, the cells were resuspended in 0.5 ml of PBS, 2% FBS. Flow cytometry involved use of FACSCalibur (BD Biosciences, San Jose, CA).

### CRISPR/Cas9-mediated genome editing

To generate FUT8-knockout (FUT8-KO) cells with the CRISPR/Cas9 system, guide RNAs (gRNAs) targeting the human FUT8 gene at exon 3 or 6 were cloned into the GeneArt CRISPR Nuclease Vector (Thermo Fisher Scientific). After sequence verification of the insert, the CRISPR/Cas9 plasmids were transfected into MDA-MB-231 or Hs578T cells. Two days after transfection, cells underwent flow cytometry-based sorting of crRNA-expressing cell populations with orange fluorescent protein expression. These crRNA-expressing cell populations were further cultured for 1 week, and FUT8-KO cells were selected by FACS analysis with LCA binding. Genomic indel modification of FUT8 in single-cell clones was assessed by PCR and sequencing.

### RNA extraction, cDNA synthesis, and RT-PCR

Total RNA was obtained from cultured cells by the TRIzol method (Thermo Fisher Scientific). First-strand cDNA synthesis with SuperScript II reverse transcriptase (Thermo Fisher Scientific) involved 5 μg RNA. The first-strand cDNA reaction was used as a template for each PCR using 2× SuperRed PCR Master Mix (BIOTOOLS, Taiwan).

### Cell migration and invasion assay

Cell migration and invasion was measured in a Boyden chamber system as described [11]. For migration assays, cells (1 × 10^5^) were placed in the upper chamber with non-coated membrane (24-well insert; 8-μm pore size; Corning Inc., Corning, NY). For invasion assays, cells (1 × 10^5^) were placed in the top chamber with Matrigel-coated membrane (24-well insert; 8-μm pore size; Corning Inc.). The total number of cells that migrated into the lower chamber was counted after 16 h of incubation at 37 °C with 5% CO_2_. Cells that migrated to the lower surface of the filter were stained with 0.5% crystal violet, examined by bright field microscopy, and photographed. Crystal violet was then dissolved with ethanol, and absorbance was read at 570 nm. Values for migration/invasion are expressed as the average number of OD570 per assay.

### Cell-based reporter assay

Cell-based IL-6 measurement was initially performed according to the manufacturer’s protocol (InvivoGen, San Diego, CA). Briefly, HEK-Blue cells were seeded at 50,000 cells per well into black clear-bottomed 96-well plates in 180 µL DMEM with 10% FBS, then incubated for 24 h with 20 µL IL-6 sample or control. A 20-µL amount of the cell culture medium was then transferred to wells of a 96-well plate containing 180 µL of QUANTI-Blue detection medium (InvivoGen) and incubated for 1–3 h. Color change indicative of secreted embryonic alkaline phosphatase (SEAP) levels was determined by using a plate reader at 620–655 nm.

### SILAC-based glycoproteomic analysis of core fucosylated proteins

Parental MDA-MB-231 or Hs578T cells were cultured in ‘light’ SILAC media (12C6 14N2 l-Lysine and 12C6 14N4 l-Arginine; Lys0 and Arg0), and two independent FUT8-KO clones of each cell line were grown in ‘medium’ SILAC media (4,4,5,5-D4 l-Lysine and 13C6 14N4 l-Arginine; Lys4 and Arg6) or in ‘heavy’ SILAC media (13C6 15N2 l-Lysine and 12C6 15N4 l-Arginine; Lys8 and Arg10) (Thermo Fisher Scientific). After metabolic labeling, cell lysates were extracted by using RIPA buffer. The core fucosylated glycoproteins were enriched with LCA-agarose beads (Vector Laboratories). After washing with PBS, on-beads trypsin digestion followed by the *N*-deglycosylation of *N*-glycosidase F released *N*-linked deglycosylated peptides from the resin. Finally, the released peptides were desalted via C18 Ziptip (Millipore) before mass spectrometry [14]. The deglycosylated peptides were analyzed by using a reverse phase NanoLC/MS/MS system consisting of NanoUPLC (NanoACQUITY, Waters, Milford, MA) and a high-resolution mass spectrometer (Orbitrap Elite, Thermo Fisher Scientific). Protein identification and relative abundance quantification involved using Proteome Discoverer (v 1.4.1.14, Thermo Fisher Scientific). The MS/MS spectra were searched with the Mascot engine (v2.5, Matrix Science, Boston, MA) against the SwissProt Homo sapiens (Human) database. Quantification node (Precursor Ions Quantifier) was used for calculation of SILAC ratio for each peptide pair and enabled the ratio calculation by replacing missing quantification values with minimum intensity.

### Pathway analysis of FUT8 target glycoproteins

Ingenuity Pathway Analysis (IPA) software (QIAGEN) was used to investigate the pathways that FUT8 targeting glycoproteins in invasive breast cancer cells may be involved in. To fulfill the format requirement of IPA, the average ratio of the intensity of heavy/light and medium/light for each target FUT8 target protein identified in MDA-MB-231 or Hs578T cells was calculated and transformed into fold change (= − 1/average ratio). When the data were imported into IPA, the gene name provided by proteomics analysis was assigned as the ID and mapped by Gene Symbol; the transformed fold change was assigned as Expression Fold Change. Individual datasets underwent core analysis including enrichment of canonical pathways, diseases, functions, upstream regulators, and networks. Enrichment of diseases and functions was summarized into categories based on molecular and cellular functions and ranked by *p* value.

Similar data transformation and core analysis was performed for the 140 FUT8 target proteins commonly identified in the two cell lines. Upstream regulator analysis is a tool that analyzes connections to target proteins via coordinator expression to identify potential upstream regulators including transcription factors and any gene or small molecule that was observed experimentally to affect gene expression in direct or indirect ways [15]. The *Z* score determines whether an upstream regulator has significantly more ‘activated’ predictions than ‘inhibited’ predictions (*z* > 0) or vice versa (*z* < 0). Six regulators showing activation *z* score > 2 or < − 2 and thus predicted to be significantly activated or inhibited in cells with FUT8 knockout were selected for constructing pathways linking FUT8 target proteins to functional outcomes. With the FUT8 target proteins as mediators, the pathways regulated by FUT8 core fucosylation and responsible for phenotypical outcomes were revealed.

### Identification of *N*-glycopeptides

Purified proteins were reduced with 10 mM dithiothreitol at 60 °C for 1 h, alkylated with 50 mM iodoacetamide in 25 mM ammonium bicarbonate buffer for 45 min in the dark at room temperature, then treated overnight with sequencing grade trypsin at an enzyme-to-substrate ratio of 1:50 at 37 °C. The digested products were then diluted with formic acid to a final concentration with 0.1% and further cleaned up by using ZipTip C18 (Millipore) before LC–MS/MS analysis. The peptide mixture was analyzed by nanoLC–MS/MS on a Tribrid Mass Spectrometer (Orbitrap Fusion Lumos, Thermo Fisher Scientific) coupled to an Easy-nLC 1200 System (Thermo Fisher Scientific). An HCD-product dependent-EThcD (HCD-pd-EThcD) workflow was used to additionally trigger EThcD events upon detecting signature glycan oxonium ions (m/z 204.0867, 138.0545, and 366.1396) during HCD fragmentation [16]. For glycopeptide identification, the MS raw data were searched by using the Byonic algorithm (v2.16.11, Protein Metrics). Glycan was searched with the default mammalian *N*-glycan database. To ensure high data quality, only glycopeptides with Byonic score > 200 were used for further analysis. The most likely glycan was predicted by using GlycoWorkbench (v2.1) from the glycan mass of the Byonic result, and the identified core-fucosylated glycopeptides were further confirmed by manual data inspection [17].

### Statistical analysis

Data are expressed as mean ± SD and were analyzed by paired *t* test. *p* < 0.05 was considered statistically significant.

## Results

### FUT8 knockout (KO) suppresses migration and invasiveness of MDA-MB-231 or Hs578T breast carcinoma cells

**Fig. 1:**
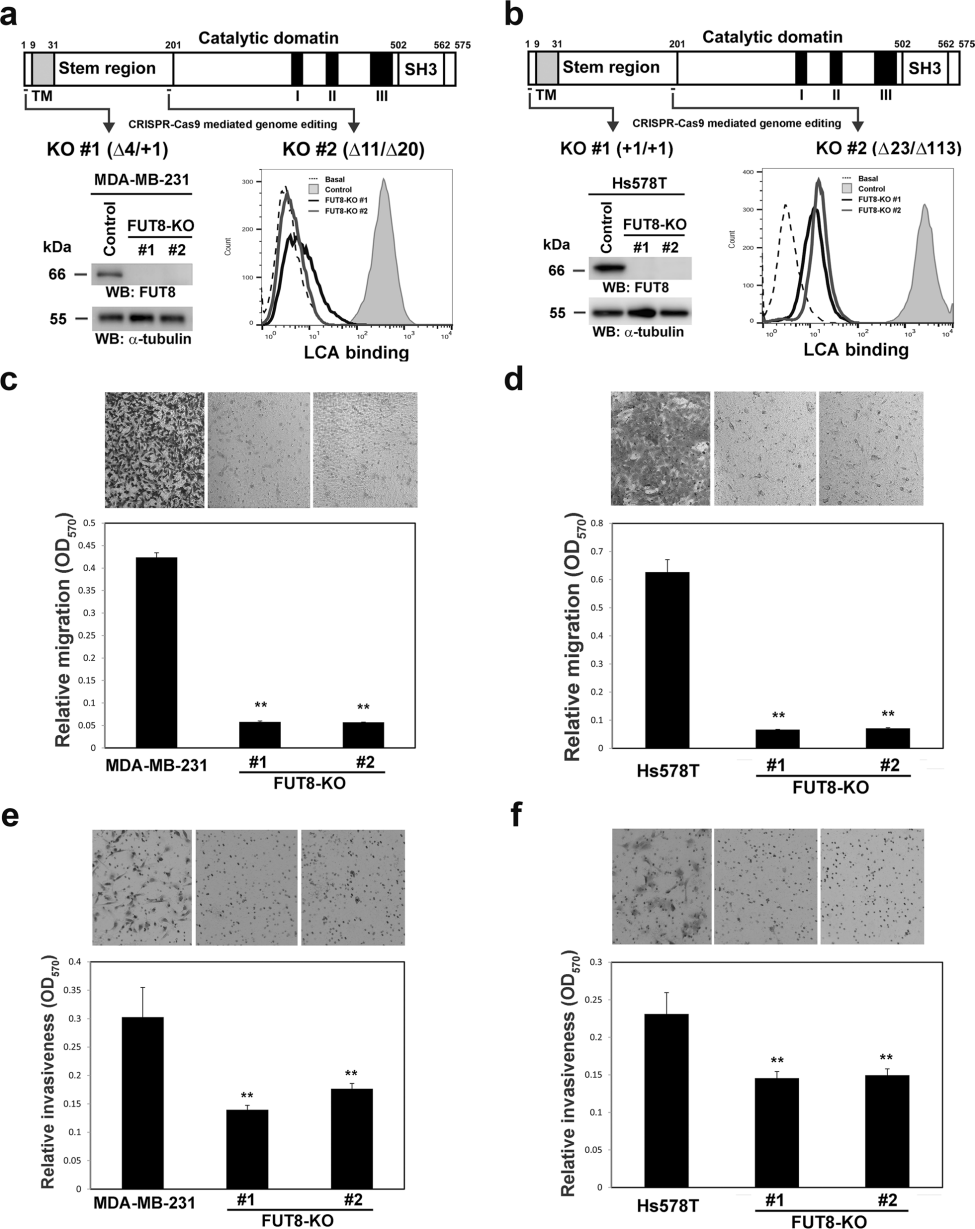
Knockout of FUT8 suppresses the invasive ability of two highly metastatic breast carcinoma cell lines. **a** and **b** Establishment of FUT8-knockout (KO) cells by CRISPR-Cas9-mediated genome editing. Protein domain organization of FTU8 is depicted on the top of each panel. TM, transmembrane domain. Two independent CRISPR-Cas9 clones targeting exon 3 or 6 of FUT8 were established (KO#1 or #2) in two invasive breast cancer cells, MDA-MB-231 (**a**) and Hs578T (**b**). Insertion (+) or deletion (Δ) mutations of each clone were verified by sequencing and are shown in parentheses. Inactivation of FUT8 gene and consequent loss of core fucosylation were validated by western blot analysis (lower-left panel) and core-specific *Lens culinaris* agglutinin (LCA) binding assay (lower-right panel). kDa, kiloDalton. Cell migration (**c**, **d**) and invasiveness (**e**, **f**) of control and FUT8-KO MDA-MB-231 (**c**, **e**) and Hs578T cells (**d**, **f**) were measured by Transwell assay with (invasion assay) and without Matrigel coating (migration assay). Data are mean ± SD. **p < 0.01, FUT8-KO versus parental cells. *OD* optical density

FUT8 is overexpressed in many human cancers and has been found associated with multiple cancer-related processes [5, 10, 14, 16, 17]. Our previous study showed that upregulated FUT8 during the TGF-β–promoted epithelial–mesenchymal transition (EMT) represents a pathological feed-forward regulatory mechanism linking core fucosylation with TGF-β receptor signaling to accelerate the EMT and promote breast cancer metastasis [11]. To search for additional FUT8 target glycoproteins and their downstream signaling during breast cancer progression, we used stable isotope labeling by amino acids in cell culture (SILAC)-based quantitative glycoproteomics. We identified glycoproteins differentially expressed in core fucose-specific lectin *Lens culinaris* agglutinin (LCA)-enriched fractions from aggressive breast cancer cells and compared these to their FUT8-KO counterparts. We first established FUT8-KO cell lines from two highly invasive breast cancer cell lines, MDA-MB-231 and Hs578T, by using the CRISPR-Cas9 system [18]. We selected two independent CRISPR sites targeting exons 3 and 6 of *FUT8* (Fig. 1). After transfecting the FUT8-targeting CRISPR/Cas9 plasmids into MDA-MB-231 (Fig. 1a) or Hs578T (Fig. 1b) cells, individual FUT8-kncockout cell clones were isolated by cell sorting, and the CRISPR/Cas9-mediated insertion or deletion mutations were verified at both genomic DNA and mRNA levels (Fig. 1a, b, upper panel), as was the protein level by western blot analysis (Fig. 1a, b, lower-left panel) and the consequent loss of core fucosylation at the cell surface by LCA binding assay (Fig. 1a, b, lower-right panel).

Cell migration and invasion are the critical steps in tumor progression and metastasis. In agreement with our previous report [11], genetic ablation of *FUT8* significantly reduced the migratory and invasive ability of MDA-MB-231 (Fig. 1c, e) and Hs578T cells (Fig. 1d, f), respectively.

### Identification and functional signaling pathway analyses of FUT8 targets

**Fig. 2:**
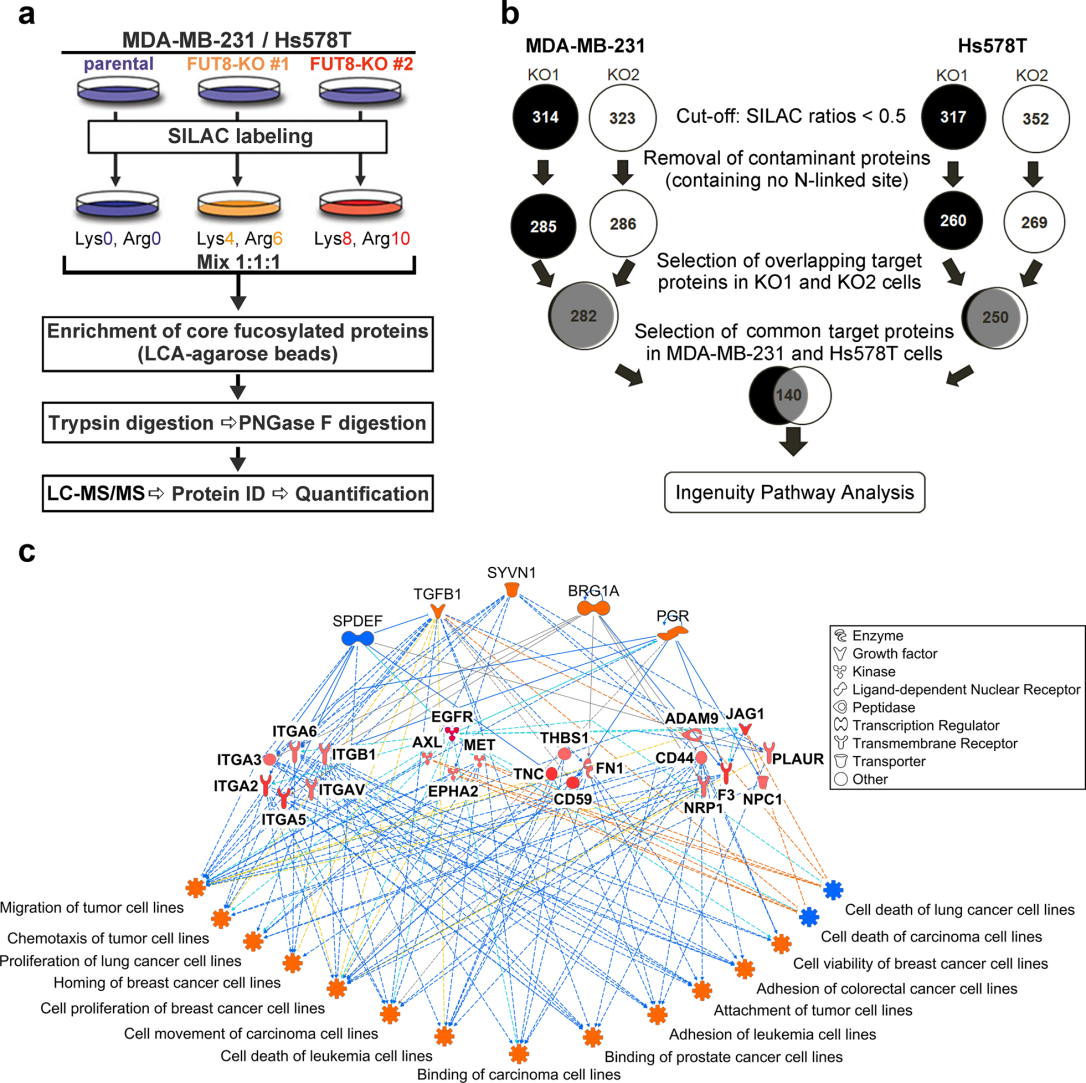
Quantitative glycoproteomics analysis. **a** Workflow of SILAC-based quantitative glycoproteomics in two highly invasive breast cancer cell lines (MDA-MB-231 and Hs578T). **b** Identification of core fucosylated glycoproteins. Using 0.5 as the threshold ratio, 282 and 250 candidate core-fucosylated glycoproteins were identified in MDA-MB-231 and Hs578T cells, respectively: 140 were common to both cell lines. **c** Functional network analysis of identified core fucosylated glycoproteins. Functional networks based on IPA predicted the activation state and subsequent effect on cellular functionality. Upstream regulators are in the top tier, and functions are in the bottom tier. FUT8 target proteins are in the middle tier. Orange lines indicate activation and blue lines suppression

To identify novel FUT8 target glycoproteins and their signaling pathways involved in breast cancer cell invasiveness, we used SILAC-based quantitative proteomic analyses to measure the differences in the core-fucosylated proteins between parental and FUT8-KO MDA-MB-231 or Hs578T cells. Parental MDA-MB-231 and Hs578T cells were cultured in ‘light’ SILAC media, whereas two independent FUT8-KO clones of each cell lines were grown in ‘medium’ SILAC media or in ‘heavy’ SILAC media (Fig. 2a). Cell lysates from the three SILAC-labeled cell populations were mixed in equal amounts. Core-fucosylated proteins were enriched by using fucose-specific LCA agarose beads. In line with the common approach for SILAC analysis, we set the threshold for downregulated proteins at 2.0-fold (SILAC ratio < 0.5). After removing apparent contaminated proteins (known to lack consensus *N*-linked glycosites), we reproducibly identified 282 or 250 target glycoproteins expressed in the invasive breast cancer MDA-MB-231 or Hs578T cells, respectively. We focused further on 140 core fucosylated proteins common to the two lines (Fig. 2b and Additional file 1: Table S1). Of note, we identified > 98% or 93% of FUT8 targets in two independent KO clones derived from these two lines, which demonstrates the sensitivity and reproducibility of our quantitative proteomics (SILAC) method.

To investigate the most enriched biological functions and molecular networks of FUT8 targets in invasive breast cancer cells, we used disease and function analysis of the two sets of FUT8 target glycoproteins with QIAGEN Ingenuity Pathway Analysis (IPA) software. Of note, the top 10 categories of molecular and cellular functions identified by these FUT8 targets from the two breast cancer cell lines seemed completely identical although in slightly different order (Additional file 2: Fig. S1a and b), which suggests that FUT8 indeed acts as a key regulator driving similar downstream pathologic processes of breast cancer (Additional file 2: Fig. S1c). These enriched pathways include cell-to-cell signaling and interaction, cellular movement, cellular growth and proliferation, cell death and survival, cell morphology, cellular assembly and organization (Additional file 2: Fig. S1a and b); the pathways modulate cell motility and cell remodeling and thus regulate cell migration and invasion. These pathways also play an important role in tumorigenesis and cancer metastasis. Another intriguing finding was that the FUT8 target glycoproteins associated with any given functional category were not necessarily identical between these two breast cancer cell lines, yet they are involved in the same molecular and cellular functions (Additional file 3: Fig. S2a–j). These results suggest that upregulated FUT8 plays an important regulatory role during breast cancer progression, which leads to core fucosylation on *N*-glycans of a variety of membrane proteins, thereby affecting cellular adhesion, migration, receptor signaling function, or interaction with other cells and substrates in a highly complex but coordinated manner.

In addition, we used regulator effects analysis of the common 140 FUT8 target glycoproteins to predict how FUT8 might increase or decrease phenotypic or functional outcomes downstream. Upstream regulator analysis is a novel IPA functional tool that can be used to analyze connections to target proteins via coordinator expression, identifying potential upstream regulators including transcription factors and any gene or small molecule that was observed experimentally to affect gene expression directly or indirectly [19]. Apart from the transcription factors including synoviolin 1 (SYVN1), transcription activator BRG1 (BRG1A), progesterone receptor (PGR), and SAM pointed domain-containing ETS transcription factor (SPDEF), TGF-β1 was also predicted to be an upstream regulator (Fig. 2c). This finding globally agrees with our previous report showing a positive feed-forward mechanism of FUT8-mediated receptor core fucosylation that enhances TGF-β signaling and the EMT, thus stimulating breast cancer cell invasion and metastasis [11]. A similar approach, regulator effect network analysis in IPA, was also used to generate connectivity networks predicting downstream functional effects and phenotypes [19]. The enriched functional networks produced included cell viability and cell proliferation of breast cancer cell lines as well as adhesion, cell movement and chemotaxis migration of many other types of cancer cells including leukemic, lung, prostate, or colorectal cancer cells (Fig. 2c).

**Fig. 3:**
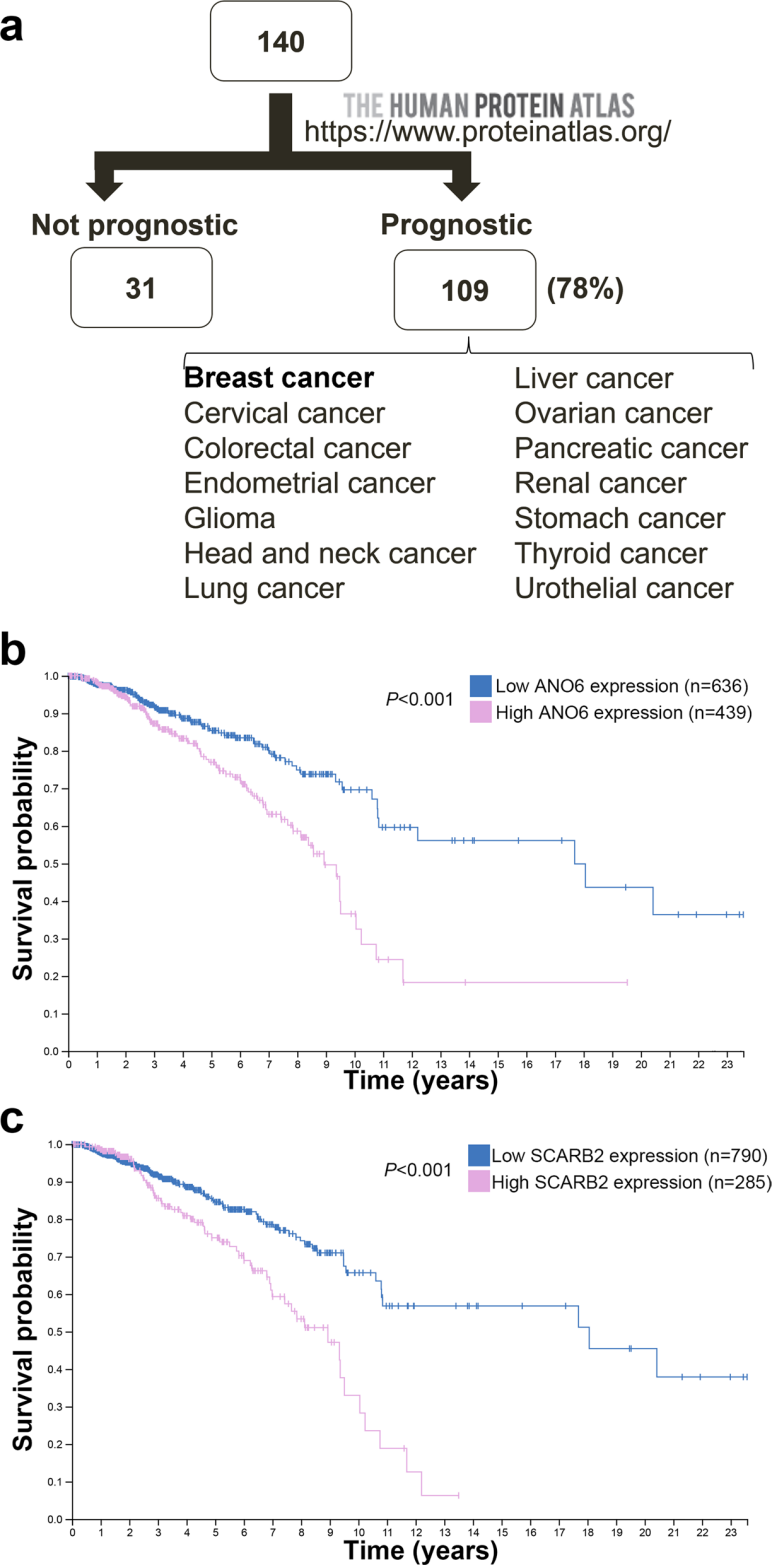
Prognostic role of identified FUT8 targets. Possible prognostic values of 140 FUT8 targets related to expression in 17 different human cancer types were assessed by using The Human Protein Atlas database. Kaplan–Meier plots are presented in the Human Protein Atlas, and genes with significantly high expression (*p* < 0.001) associated with patient survival were defined as prognostic genes. **a** In all, 109 of 140 FUT8 targets showed clinical prognostic values in a number of cancer types in The Human Protein Atlas. **b** Prognostic value of ANO6 expression in breast cancer patients (The Human Protein Atlas at https://www.proteinatlas.org). **c** Prognostic value of SCARB2 expression in breast cancer patients (The Human Protein Atlas at https://www.proteinatlas.org)

To further explore the pathological implications and potential prognostic values, these 140 FUT8 targets were further assessed by using an interactive open-access database, The Human Protein Atlas database (http://www.proteinatlas.org). The database contains information about protein localization in normal organs as well as detection of these proteins in 17 different cancer types. Correlation analyses based on their expression in cancer tissue and clinical outcome for cancer patients revealed that many (109/140, 78%) of the FUT8 targets had clinical prognostic values for breast cancer and also other cancer types: cervical, colorectal, endometrial, glioma, head and neck, lung, liver, ovarian, pancreatic, renal, stomach, thyroid, and urothelial cancers (Fig. 3a and Additional file 4: Table S2). For instance, the Kaplan–Meier survival analysis revealed that breast cancer patients with high expression of ANO6 (Anoctamin 6, a multi-pass transmembrane protein that is a regulator of phospholipid scrambling) [20] or SCARB2 (Scavenger receptor class B member 2, a type III glycoprotein that is located primarily in limiting membranes of lysosomes/endosomes and participates in membrane transportation and the reorganization of endosomal/lysosomal compartment) [15] had a shortened survival compared to those in the low expression group (Fig. 3b, c). Together, these results further verify that the FUT8 target glycoproteins we identified indeed have broader pathological involvement in cancer types beyond breast cancer.

### Validation of candidate FUT8 target proteins

**Fig. 4:**
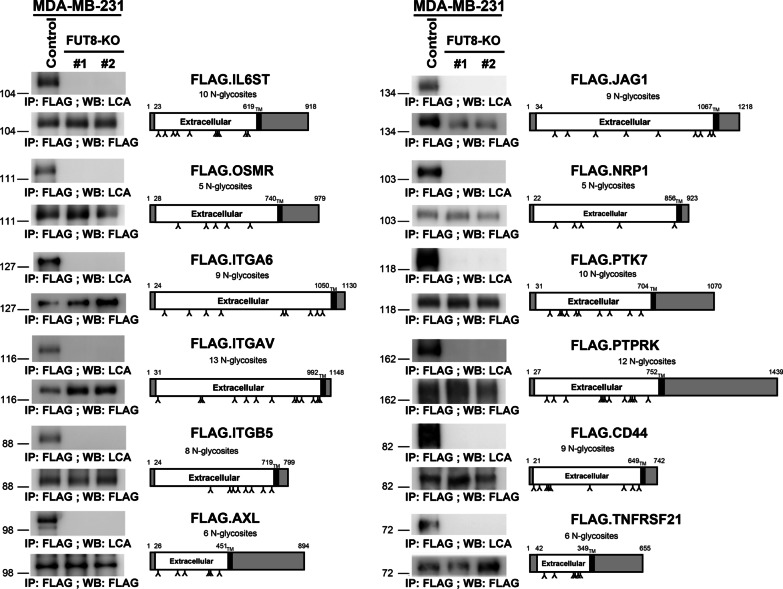
Validation of the SILAC results of identified glycoproteins. Core fucosylation of the selected proteins was eliminated by FUT8 knockout (KO). Recombinant proteins in the control or two FUT8-KO MDA-MB-231 cell lines were probed with biotinylated LCA, then detected with streptavidin-conjugated horseradish peroxidase. Protein domain organization of these selected proteins was depicted according to the Uniprot database (https://www.uniprot.org); potential N-linked glycosylation sites are marked. *TM* transmembrane domain

**Fig. 5:**
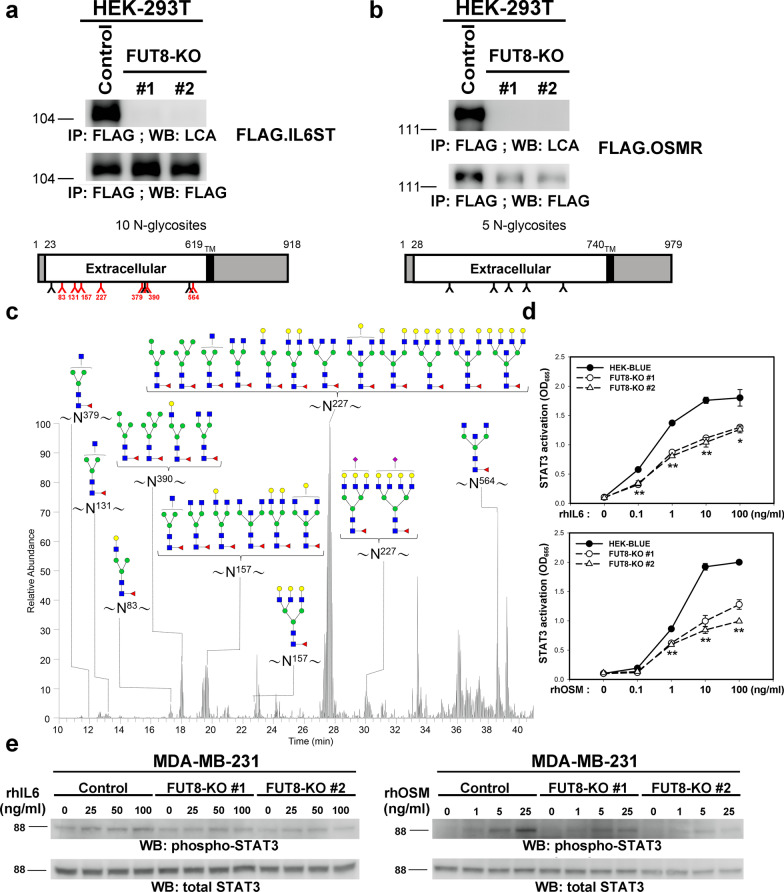
FUT8 modulates core fucosylation of IL6ST protein and its downstream signaling. **a** and **b** IL6ST and OSM receptor (OSMR) proteins core fucosylated by FUT8 in HEK-293 T cells. Recombinant IL6ST or OSMR proteins in control or two FUT8-KO 293 T cell lines were probed with biotinylated LCA, then detected with streptavidin-conjugated horseradish peroxidase. Protein domain organization of these selected proteins is illustrated according to Uniprot data (http://uniprot.org). Amino acid number and potential *N*-linked glycosylation sites are marked on the upper and lower side, respectively. Core-fucosylated sites confirmed by LC–MS/MS analyses are in red. TM, transmembrane domain. **c** Core-fucosylation sites of IL6ST protein. Shows LC–MS/MS summed extracted ion chromatogram (XIC) of identified core-fucosylated glycopeptides for IL6ST. The number on asparagine (N) indicates the position in the protein sequence. **d** FUT8 KO impaired IL-6 and OSM signaling in STAT3 reporter HEK cells. Parental or FUT8-KO HEK-Blue™ IL-6 cells (InvivoGen) were treated with recombinant IL-6 (upper) or OSM (lower). Levels of STAT3-inducible secreted embryonic alkaline phosphatase (SEAP) indicating STAT3 activity were monitored by using QUANTI-Blue. Data are mean ± SD. **p < 0.01, *p < 0.05, FUT8-KO #1 or FUT8-KO#2 versus parental HEK-Blue™ IL-6 cells. **e** FUT8 KO impaired IL-6 and OSM signaling in MDA-MB-231 cells. Control and FUT8-KO MDA-MB-231 cells were treated with the indicated concentrations of recombinant IL-6 (left panel) or OSM (right panel) for 15 min. Cell lysates underwent western blot analysis with antibody for phosphorylated STAT3 or total STAT3

From the 140 common FUT8 glycoproteins, we further selected 19 membrane or receptor proteins with potential biological functions in cancer cell growth, proliferation, adhesion, or migration for further validation, including integrins (ITGA2, ITGA3, ITGA5, ITGA6, ITGAV, ITGB1, and ITGB5), cell-surface proteins (PTK7, TNFRSF21, JAG1, THBD, and CD44) and signaling receptors (AXL, CDCP1, NPR1, PTPRK, EGFR, OSMR, and IL6ST) (Additional file 5: Fig. S3, Figs. 5a, b and 8a). We first examined whether these targets are truly modified by FUT8-catalyzed core fucosylation by using core fucose-specific LCA lectin blot analysis. When overexpressed in control HEK-293 T cells, all FUT8 candidates were indeed core-fucosylated and detected by LCA blotting, which was completely abolished in FUT8-deficient HEK-293 T cell lines (Additional file 5: Fig. S3, Figs. 5a, b and 8a). Importantly, we also performed a negative control by using another lectin RCA I (*Ricinus communis* agglutinin I) that recognizes terminal galactose or WGA (Wheat germ agglutinin) that binds oligosaccharides containing terminal *N*-acetylglucosamine. While the FLAG-tagged IL6ST is not core fucosylated (determined by LCA lectin) by FUT8, addition of the terminal galactose (detected by RCA I lectin) or *N*-acetylglucosamine (detected by WGA) is still glycosylated in the FUT8-KO cells (Additional file 6: Fig. S4). In agreement with these findings, we further confirmed 12 of these candidate FUT8 target proteins including IL6ST, OSMR, ITGA6, ITGAV, ITGB5, AXL, JAG1, NRP1, PTK7, PTPRK, CD44, or TNFRSF21 that are truly core fucosylated in breast carcinoma MDA-MB-231 cells (Fig. 4). Thus, these results validate our overall quantitative glycoproteomics approach in identifying genuine FUT8 targets and also verify that these glycoproteins are authentically modified by core fucosylation.

### Functional characterization of novel FUT8 core-fucosylated proteins conferring pro-invasive/pro-metastatic behavior to breast cancer cells

To investigate the roles of core fucosylation of the newly identified FUT8 target proteins, we selected IL6ST and integrin αvβ5 for further study. The cytokine receptor IL6ST is the common signaling subunit for the interleukin (IL)-6 family of cytokines including IL-6, OSM, leukemia inhibitory factor, cardiotrophin-1, and ciliary neurotrophic factor. Binding of IL-6 to IL-6 receptor (IL-6R) induces homodimerization and recruitment of IL6ST, which activates downstream signaling [21]. OSM activates a complex of OSM receptor (OSMR) and IL6ST. Numerous studies have reported that IL6ST plays a role in tumor progression and metastasis [22–24]. Previous study showed that IL-6 acts as an inducer of an EMT phenotype in breast cancer cells, which implicates its potential to promote breast cancer metastasis [25]. In addition, OSM can promote an EMT/cancer stem cell-like phenotype in breast cancer and enhance migration [26].

**Fig. 6:**
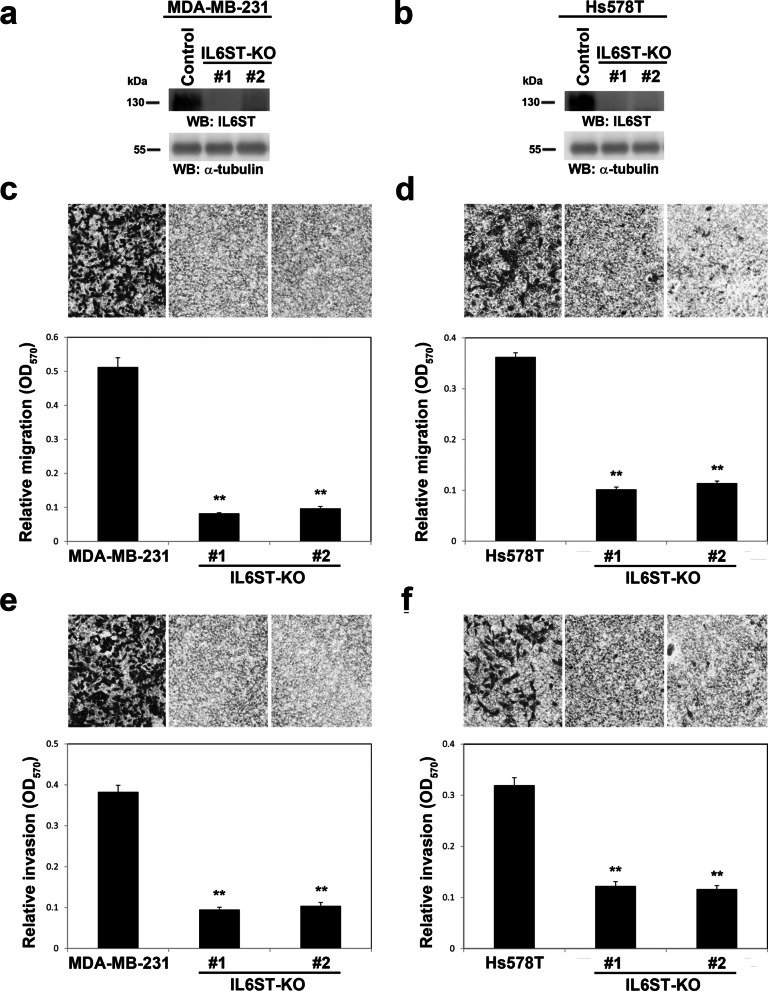
Establishment of IL6ST-knockout cells by CRISPR-Cas9-mediated genome editing. Two independent CRISPR-Cas9 clones targeting exon 3 and 8 of IL6ST were established (KO#1 or #2) in two invasive breast cancer cells, MDA-MB-231 (**a**) or Hs578T (**b**), respectively. Depletion of IL6ST gene were validated by western blot. kDa, kiloDalton. Cell migration (**c**, **d**) and invasiveness (**e**, **f**) of control and IL6ST-KO MDA-MB-231 (**c**, **e**) and Hs578T cells (**d**, **f**) by Transwell assay with (invasion assay) and without Matrigel coating (migration assay). Data are mean ± SD. **p < 0.01, IL6ST-KO versus parental cells. *OD* optical density

**Fig. 7:**
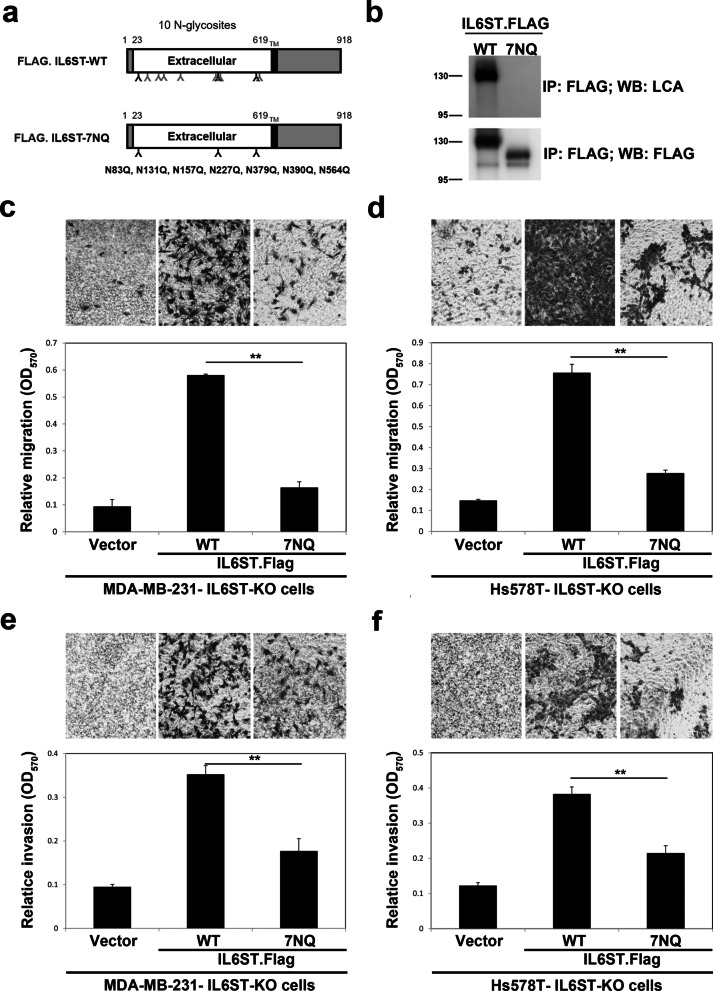
Core fucosylation sites of IL6ST and its biological function. **a** Schematic diagram of human IL6ST wild type (WT) and its core fucosylation site mutant IL6ST-7NQ. TM, transmembrane domain. **b** Western blot and LCA blot analysis of immunoprecipitated FLAG.IL6ST-WT and FLAG.IL6ST-7NQ. **c**–**f** Effect of IL6ST core fucosylation site mutant on the migratory and invasive ability of breast cancer cells. Cell migration (**c**, **d**) and invasiveness (**e**, **f**) of IL6ST-KO MDA-MB-231 (**c**, **e**) and Hs578T cells (**d**, **f**) re-expressing FLAG.IL6ST-WT or FLAG.IL6ST-7NQ were measured by Transwell assay with (invasion assay) and without Matrigel coating (migration assay). Data are mean ± SD. **p < 0.01. *OD* optical density

When overexpressed in control MDA-MB-231 or HEK-293 T cells, IL6ST and OSMR proteins were core-fucosylated, but their core fucosylation was completely absent in FUT8-KO counterpart cell lines (Figs. 4 and 5a, b). To further identify the detailed glycan structure, we analyzed the digested glycopeptides of purified IL6ST expressed in HEK-293 T cells by LC–MS/MS. We identified glycopeptides containing N83, N131, N157, N227, N379, N390, and N564 carrying core fucose (Fig. 5c and Additional file 7: Table S3), but this core fucosylation was not observed in protein samples from FUT8-KO cells (data not shown). We further determined whether ablation of IL6ST core fucosylation could affect the IL-6 signaling pathway. To this end, we evaluated whether FUT8 KO affects IL-6-stimulated transcriptional activity in IL-6 sensor cells, HEK-Blue™ IL-6 cells, which were generated by stable transfection of HEK-293 cells with the human IL-6R gene and a STAT3-inducible secreted embryonic alkaline phosphatase (SEAP) reporter gene [27]. Upon IL-6 stimulation, HEK-Blue™ IL-6 cells activated STAT3 and secretion of SEAP (Fig. 5d). Levels of STAT3-induced SEAP can be readily monitored by using QUANTI-Blue™. FUT8-KO cells showed diminished transcriptional activity induced by IL-6 or OSM. Moreover, IL-6 or OSM-activated STAT3, quantified by its phosphorylation, was significantly reduced in FUT8-KO MDA-MB-231 cells as compared with control cells (Fig. 5e). Next, we sought to determine whether core fucosylation of IL6ST is critical for its pro-invasive ability. We first established IL6T-KO cell lines from MDA-MB-231 (Fig. 6a) and Hs578T (Fig. 6b) by using the CRISPR-Cas9 approach. Depletion of IL6ST significantly reduced the migratory and invasive ability of MDA-MB-231 (Fig. 6c, e) and Hs578T cells (Fig. 6d, f), respectively. We then generated a mutant form IL6ST-7NQ (substitution of asparagines of identified core fucosylation site N83, N131, N157, N227, N379, N390, and N564 with glutamines; Fig. 7a). Using LCA blotting, we confirmed the loss of core fucosylation in IL6ST-7NQ mutants (Fig. 7b). IL6ST or IL6ST-7NQ mutant were reconstituted back in IL6ST-KO MDA-MB-231 or Hs578T cells to determine whether core fucosylation of IL6ST affect cell migration and invasion, respectively. While re-expression of wild-type IL6ST can rescue the migratory and invasive ability of IL6ST-KO MDA-MB-231 (Fig. 7c, e) and Hs578T cells (Fig. 7d, f), IL6ST-7NQ mutant fails to restore migration and invasion of IL6ST-KO cells. Together, these results suggest that core fucosylation of IL6ST is critical for its ability to transmit pro-invasive and pro-metastatic IL-6 or OSM signaling.

**Fig. 8:**
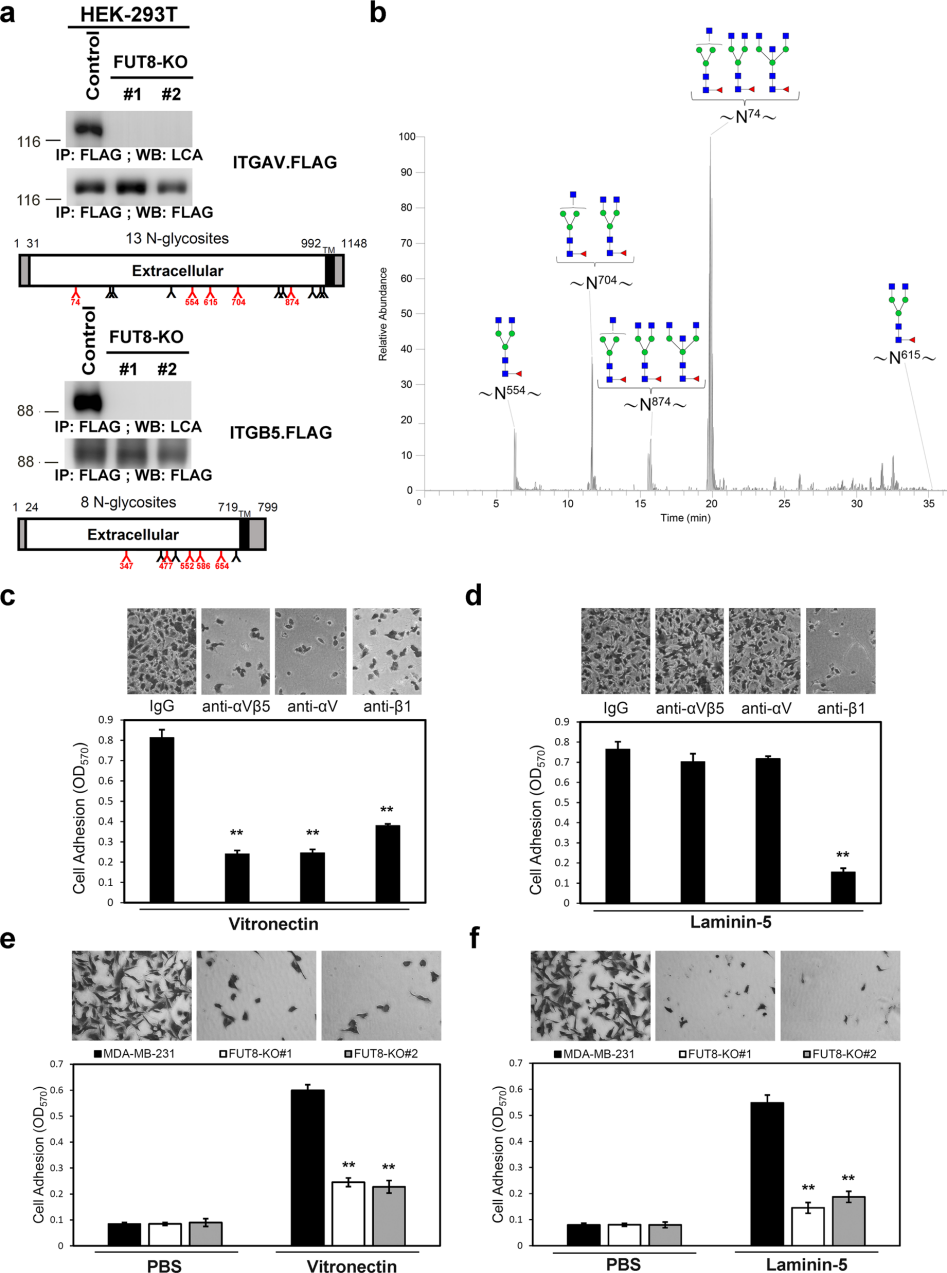
FUT8 modulates core fucosylation and adhesive capability of αvβ5 integrins. **a** Integrins αv and β5 were core fucosylated by FUT8 in HEK-293 T cells. Recombinant integrin αv (upper panel) and integrin β5 (lower panel) in the control or two FUT8-KO 293 T cell lines were probed with biotinylated LCA, then detected with streptavidin-conjugated horseradish peroxidase. Protein domain organization of these selected proteins are depicted according to Uniprot data. Amino acid number and potential N-linked glycosites are marked on the upper and lower side, respectively. Core-fucosylated sites verified by LC–MS/MS analysis are in red. TM, transmembrane domain. **b** Core fucosylation of integrin αv. LC–MS/MS summed extracted ion chromatogram (XIC) of identified core-fucosylated glycopeptides for integrin αv. The number on asparagine (N) indicates the position in the protein sequence. **c** and **d** Functional blocking antibodies abolished cell adhesion to vitronectin and laminin-5 in MDA-MB-231 cells. Plates were pre-coated with 5 μg/ml vitronectin (**c**) or laminin-5 (**d**). MDA-MB-231 cells were pre-incubated with anti-integrin antibodies (10 μg/ml) before cells were placed on coated plates for 10 min at 37 °C. Adherent cells were quantified after fixation and stained with crystal violet. Data are mean ± SD. **p < 0.01 compare with control IgG. **e** and **f** FUT8 KO reduced cell adhesion to vitronectin and laminin-5 in MDA-MB-231 cells. Parental or FUT8-KO MDA-MB-231 cells were allowed to attach to vitronectn-coated (**e**) or laminin-5-coated (**f**) plates. Adherent cells were quantified after fixation and stained with crystal violet. Data are mean ± SD. **p < 0.01, FUT8-KO versus parental cells

Other FUT8 target proteins that drew our attention were integrins. Integrins are a family of 24 transmembrane heterodimers generated from a combination of 18 α integrin and 8 β integrin subunits. Much literature has reported the roles of integrins in human cancer progression [28, 29]. Altered integrin expression during breast cancer progression has been reported in several studies [30, 31]. Apart from their expression, the cellular function of integrins could also be regulated at post-translational levels. For example, core fucosylation is required for the function of integrin α3β1-mediated cell migration and interaction of α4β1 integrin and vascular cell adhesion molecule 1 [13, 32]. Nevertheless, the impact of core fucosylation on other integrins during breast cancer progression remains largely unknown. The introduction of dominant-negative E-cadherin increased migration on vitronectin due to increased activity of αvβ5 integrins [33]. Integrin αvβ5 has a role in cell invasion. Some studies suggested that αvβ5-dependent breast cancer migration may be partly regulated by urokinase [34]. Therefore, we further focused on integrin αvβ5 and validated that integrins αv or β5 subunit was indeed core-fucosylated by LCA blotting (Figs. 4 and 8a). In addition, glycan profiling of purified αv or β5 subunit identified glycopeptides containing *N*-glycosylation sites at N74, N554, N615, N704, N874 of integrin αv. Sites at N347, N 477, N552, N586, N654 of integrin β5 were verified as carrying core fucose (Fig. 8b, Additional file 7: Table S3 and Additional file 8: Fig. S5). Core fucosylation of these sites was completely abolished in samples from FUT8-KO cells (data not shown).

Integrins bind to extracellular matrix components and provide the traction necessary for cell motility and invasion that are critical for cancer progression and metastasis. For example, the interaction of α3β1 integrin with the ligand laminin-5 promotes the migration and invasion of malignant glioma and melanoma cells [35]. The vitronectin-binding receptor αvβ5 integrin has a crucial role in breast malignancy [36]. Metastatic breast cancer cells showed increased expression of αvβ5 integrin. In addition, αvβ5 integrin promotes cancer metastasis via activation of extracellular-signal-regulated kinase 5 [37]. We further investigated a functional combination of integrins in MDA-MB-231 cells by using function-blocking monoclonal antibodies against αvβ5 or αv integrin. We also included function-blocking monoclonal antibodies against β1 integrins because core fucosylation is essential for the functions of α3β1 integrin [13]. The antibody against αvβ5, an αv integrin, almost completely abolished MDA-MB-231 adhesion to vitronectin but had no effect on laminin-5 binding; whereas anti-β1 integrin antibodies were more effective at preventing cell adhesion to laminin-5 (Fig. 8c, d). Thus, αvβ5 integrin likely mediates cell adhesion to vitronectin, whereas β1 likely binds to both vitronectin and laminin-5 in MDA-MB-231 cells. Next, we evaluated the adhesive ability of parental or FUT8-KO MDA-MB-231 cells on the integrin ligands vitronectin and laminin-5. As compared with parental MDA-MB-231 cells, FUT8-KO cells showed significantly reduced adhesion to vitronectin and laminin-5 (Fig. 8e, f). Collectively, these results suggest that αvβ5 integrin is a key molecule for cell adhesion on vitronectin in MDA-MB-231 cells and that core fucosylation probably regulates αvβ5 integrin-mediated cell adhesion.

## Discussion

To identify novel targets for improving new diagnostic approaches and therapeutic strategies for breast cancer, one must have a more complete understanding of breast cancer biology at the molecular level. We recently identified FUT8 as a key regulator during breast cancer metastasis [11]. However, the full spectrum of FUT8 target glycoproteins and a comprehensive network of signaling pathways orchestrated by FUT8 for breast cancer cell migration and invasion leading to metastasis remain elusive and uninvestigated. In the present study, we used loss-of-function experiments and functional proteomics with knocking out FUT8 in MDA-MB-231 and Hs578T breast cancer cells to reveal a broad list of core fucosylated target glycoproteins. Our SILAC-based comparative quantitative glycoproteomics analysis identified core-fucosylated proteins, with enrichment in membrane glycoproteins involved in cancer cell migration and invasion. The FUT8 target glycoproteins in breast cancer we identified in general agree with those in previous studies of melanoma or non-small cell lung cancer by sharing greater than 78% coverage [14, 16]. Therefore, these findings further validated the effectiveness of identifying FUT8 targets by our SILAC-based quantitative glycoproteomics approach.

Some of these FUT8 target proteins have been reported in other human cancers. For example, EGFR and MET core fucosylation can potentiate their ligand binding ability [12, 38] and thus may enhance downstream signaling to support tumor growth and metastasis in liver cancer. Integrins are critical for metastasis [39]. Deletion of *Fut8* significantly impaired integrin α3β1-mediated cell migration of mouse embryonic fibroblasts [13]. The receptor tyrosine kinase AXL has been implicated in EMT, invasion and chemo-resistance of several human cancers [40]. Moreover, aberrant glycosylation of AXL was reported to mediate tumor cell proliferation, invasion and metastasis [41]. Neuropilin-1 is a non-tyrosine kinase receptor implicated in tumor progression [42]. Growing evidence implicates a role for neuropilin-1 in promoting cancer progression by acting as a co-receptor for molecules involved in the EMT pathway and metastasis in tumor tissue such as vascular endothelial growth factor, placental growth factor, and TGF-β1 [43].

Importantly, we further verified the specific core-fucosylation sites and potential glycan composition of each site in the signaling receptor IL6ST and adhesion receptor αvβ5. We demonstrated that core fucosylation of IL6ST promoted IL-6– and OSM-stimulated cellular signaling important for breast cancer EMT and metastasis. It also promoted αvβ5 integrin-mediated cell adhesion on vitronectin in aggressive breast cancer cells. Although the precise molecular and structural basis of how core fucosylation modulates biological activities of their targets is not yet fully defined, core fucosylation of IL6ST or integrin αv/β5 subunits or even any specific residue may contribute to certain conformations optimal for receptor–ligand interactions. These conformations may facilitate signaling activity or enhance affinity between integrin and extracellular substrates. In support of this notion, a recent study used in silico modeling to demonstrate that fucosylated glycan showed more restricted movement than non-fucosylated glycan on the surface of integrin β1, possibly favoring the interaction of glycan with other molecules [44]. Nevertheless, additional investigations are required to elucidate the molecular mechanism underlying the structural and functional consequences of core fucosylation of IL6ST or αvβ5 during the EMT.

Given the critical roles of glycosylation alterations in cancer biology, strategies that reveal aberrant glycosylation of cancer cells would benefit the diagnosis and treatment of cancer. We and others have reported the association of core fucosylation with tumor progression in breast, melanoma, liver, prostate and lung cancers [11, 14, 16, 17, 38]. To date, no specific FUT8 inhibitor for core fucosylation is available. However, this inhibitor could result in severe side effects due to vital roles of core fucosylation during developmental and physiological processes. Indeed, genetic inactivation of *Fut8* led to emphysema, severe growth retardation and early postnatal lethality in mice [45]. Therefore, better therapeutic approaches may be those aimed at critical FUT8 targets and/or their downstream signaling pathways. Of note, most (> 90%) of FUT8 targets in invasive breast cancer cells are indeed druggable because they are secreted or membrane-anchored glycoproteins. For instance, to limit the above-mentioned off-target side effects, neutralizing antibodies could be developed against aberrant core fucosylation of pro-invasive/pro-metastatic glycoproteins such as integrin αvβ5 or IL6ST identified in this study or cancer growth-promoting cell surface epidermal growth factor receptor [16]. By discriminating FUT8-mediated normal physiological from pathological signaling, these might be more effective therapeutic means.

## Conclusions

In summary, this is the first report to comprehensively identify FUT8 target glycoproteins and their downstream signal networks in aggressive breast cancer carcinoma cells by using a SILAC-based quantitative glycoproteomics method. These FUT8 targets reveal a global and novel aspect of signaling pathways and networks essential for breast cancer migration, invasion, and metastasis. In addition, integrin αvβ5 and IL6ST were verified as novel glycoprotein targets of FUT8. Core fucosylation of these molecules plays important functions in breast cancer cell adhesion to vitronectin, and their responsiveness to IL-6 or OSM signaling is involved in breast cancer EMT and metastasis. Interestingly, unappreciated cellular processes such as dynamics of membrane leaflet phospholipid composition or membrane transportation and reorganization of endosomes/lysosomes regulated by core fucosylated ANO6 [20] or SCARB2 [15] potentially involved in breast cancer progression had been identified and warrant further investigations. These FUT8 glycoproteins provide a resource for further understanding molecular mechanisms underlying breast cancer invasion and metastasis and are potential targets for preventing and treating metastatic breast cancers.

## Supplementary Information

**Additional file 1: Table S1.** Core fucosylated FUT8 target proteins identified by comparative quantitative glycoproteomics.

**Additional file 2: Fig. S1.** Top 10 enriched diseases and biological functions.

**Additional file 3: Fig. S2.** Venn diagrams show the FUT8 targets involved in the functions and identified in both invasive breast carcinoma cell lines.

**Additional file 4: Table S2.** Prognostic value of FUT8 target proteins.

**Additional file 5: Fig. S3.** Validation of the SILAC results of identified glycoproteins.

**Additional file 6: Fig. S4.** Specific remove of core fucose but not terminal galactose in the FUT8-deficient cells.

**Additional file 7: Table S3.** List of identified core fucosylated glycopeptides for IL6ST, integrin αv, and integrin β5.

**Additional file 8: Fig. S5.** Core fucosylation sites of integrin β5.

## Data Availability

The datasets used or analysed during the current study are available from the corresponding author on reasonable request.

## Abbreviations

Cas9: CRISPR associated protein 9
CRISPR: Clustered regularly interspaced short palindromic repeats
EMT: Epithelial–mesenchymal transition
FUTs: Fucosyltransferases
LCA: *Lens culinaris agglutinin*
TGF-β: Transforming growth factor-β
