# Supplementary material for: Quantitative glycoproteomics analysis identifies novel FUT8 targets and signaling networks critical for breast cancer cell invasiveness
Source: Breast Cancer Res. 2022 Mar 18;24:21. doi: 10.1186/s13058-022-01513-3 (PMC8932202; doi:10.1186/s13058-022-01513-3)
Supplement: Supplementary file 2 — Additional file 2: Fig. S1. Top 10 enriched diseases and biological functions. [file 13058_2022_1513_MOESM2_ESM.pdf]

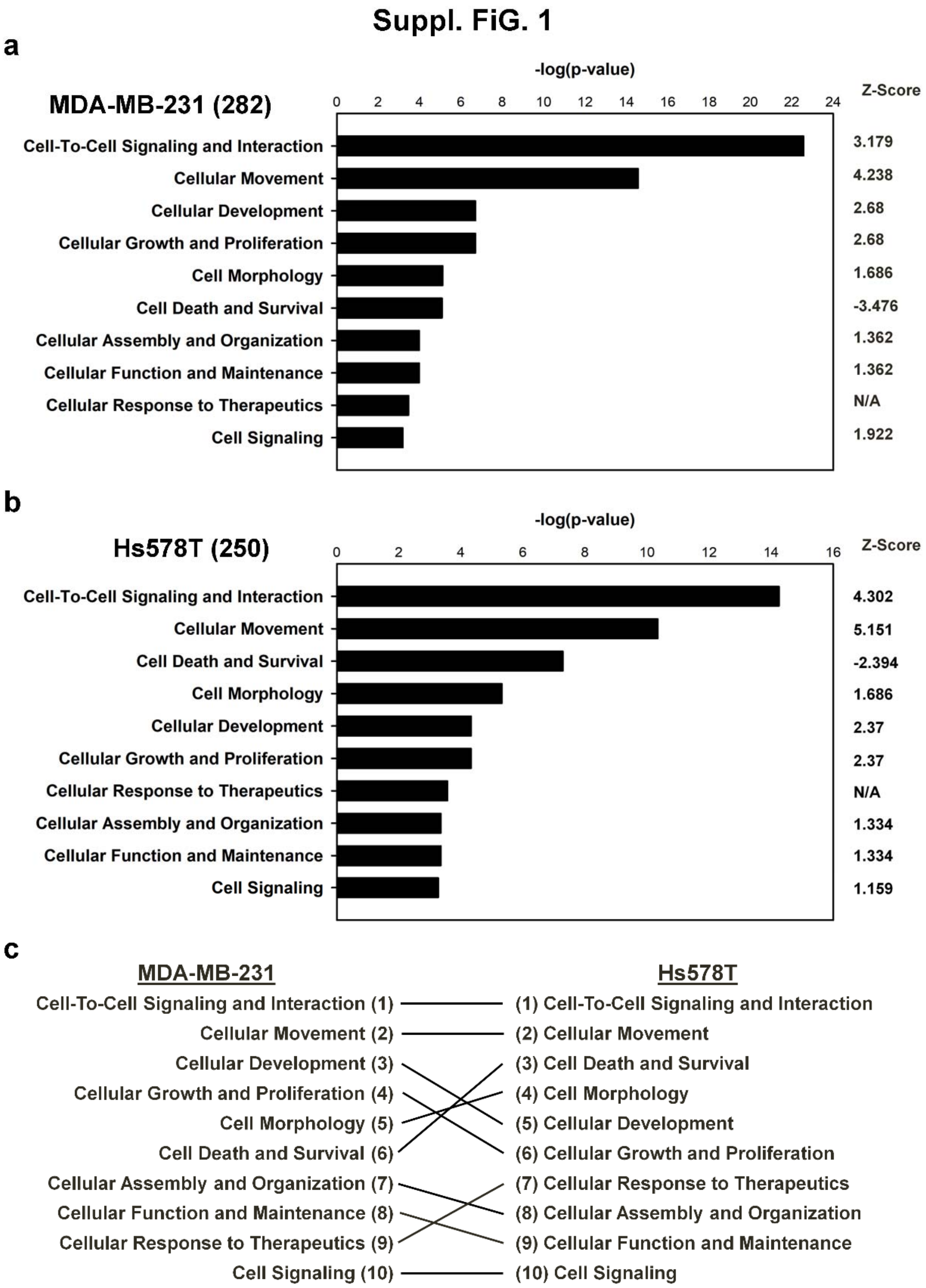

#

Figure S1. Top 10 enriched diseases and biological functions. Identified candidate FUT8 target proteins were further analyzed with IPA® software to outline the most enriched biological functions in MDA-MB-231 (a) and Hs578T (b) cells. Activation z score was calculated by the IPA software and predicted whether a specific function is increased (positive z score) or decreased (negative z score) based on the experimental datasets. (c) comparison of functions in MDA-MB-231 and Hs578T cells.
