# Supplementary material for: Quantitative glycoproteomics analysis identifies novel FUT8 targets and signaling networks critical for breast cancer cell invasiveness
Source: Breast Cancer Res. 2022 Mar 18;24:21. doi: 10.1186/s13058-022-01513-3 (PMC8932202; doi:10.1186/s13058-022-01513-3)
Supplement: Supplementary file 3 — Additional file 3: Fig. S2. Venn diagrams show the FUT8 targets involved in the functions and identified in both invasive breast carcinoma cell lines. [file 13058_2022_1513_MOESM3_ESM.pdf]

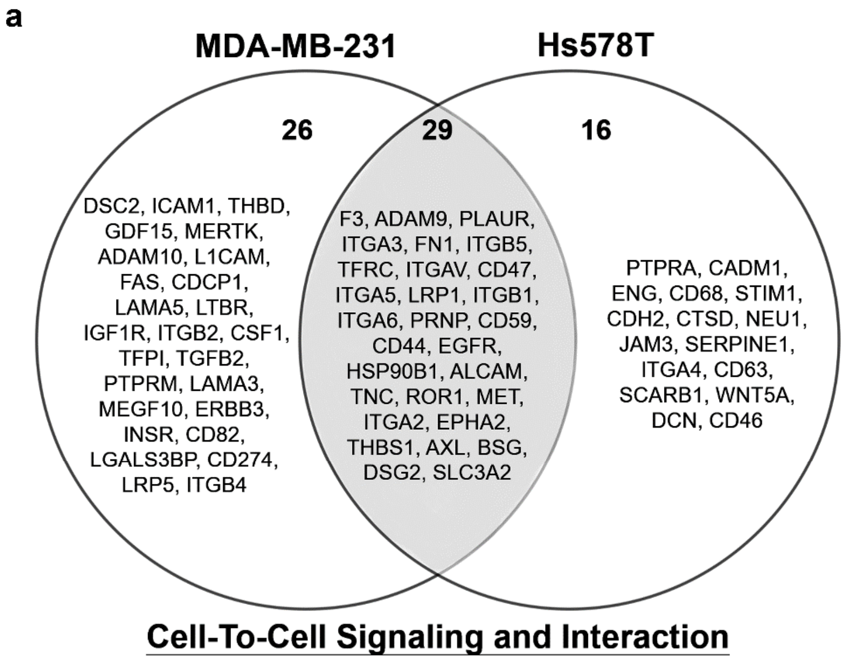
a
MDA-MB-231
Hs578T
26
29
16
DSC2, ICAM1, THBD, GDF15, MERTK, ADAM10, L1CAM, FAS, CDCP1, LAMA5, LTBR, IGF1R, ITGB2, CSF1, TFPI, TGFB2, PTPRM, LAMA3, MEGF10, ERBB3, INSR, CD82, LGALS3BP, CD274, LRP5, ITGB4
F3, ADAM9, PLAUR, ITGA3, FN1, ITGB5, TFRC, ITGAV, CD47, ITGA5, LRP1, ITGB1, ITGA6, PRNP, CD59, CD44, EGFR, HSP90B1, ALCAM, TNC, ROR1, MET, ITGA2, EPHA2, THBS1, AXL, BSG, DSG2, SLC3A2
PTPRA, CADM1, ENG, CD68, STIM1, CDH2, CTSD, NEU1, JAM3, SERPINE1, ITGA4, CD63, SCARB1, WNT5A, DCN, CD46
Cell-To-Cell Signaling and Interaction

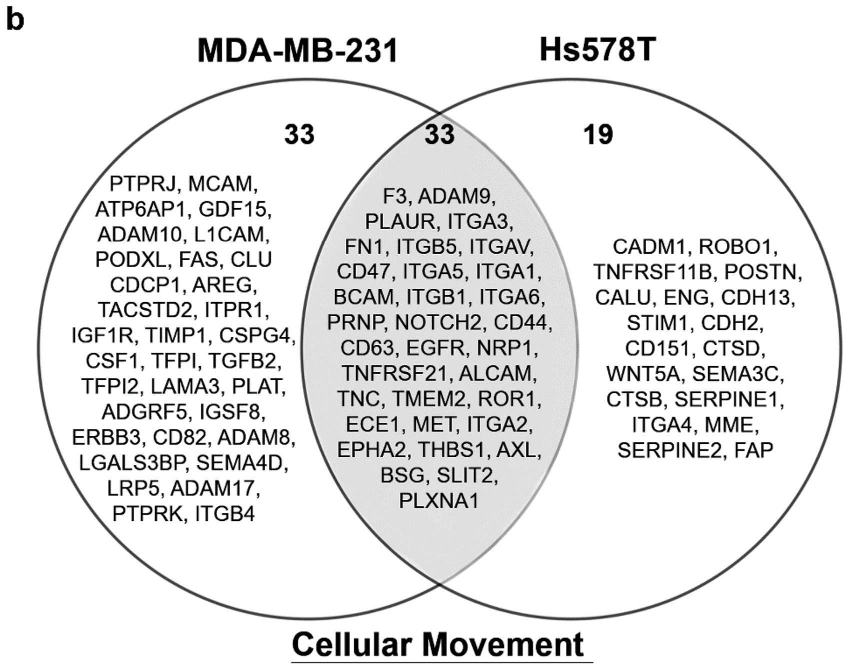
b
MDA-MB-231
Hs578T
33
33
19
PTPRJ, MCAM, ATP6AP1, GDF15, ADAM10, L1CAM, PODXL, FAS, CLU CDCP1, AREG, TACSTD2, ITPR1, IGF1R, TIMP1, CSPG4, CSF1, TFPI, TGFB2, TFPI2, LAMA3, PLAT, ADGRF5, IGSF8, ERBB3, CD82, ADAM8, LGALS3BP, SEMA4D, LRP5, ADAM17, PTPRK, ITGB4
F3, ADAM9, PLAUR, ITGA3, FN1, ITGB5, ITGAV, CD47, ITGA5, ITGA1, BCAM, ITGB1, ITGA6, PRNP, NOTCH2, CD44, CD63, EGFR, NRP1, TNFRSF21, ALCAM, TNC, TMEM2, ROR1, ECE1, MET, ITGA2, EPHA2, THBS1, AXL, BSG, SLIT2, PLXNA1
CADM1, ROBO1, TNFRSF11B, POSTN, CALU, ENG, CDH13, STIM1, CDH2, CD151, CTSD, WNT5A, SEMA3C, CTSB, SERPINE1, ITGA4, MME, SERPINE2, FAP
Cellular Movement

c

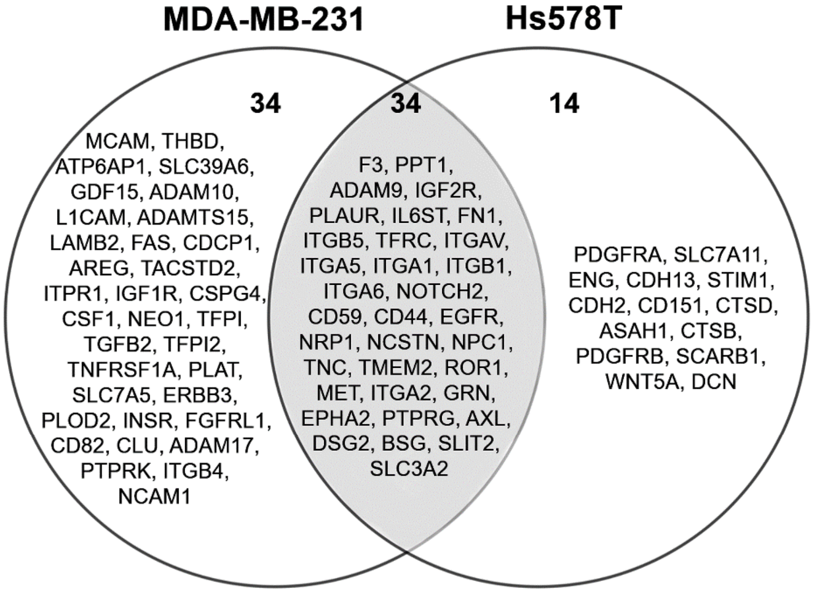

Cellular Development

d

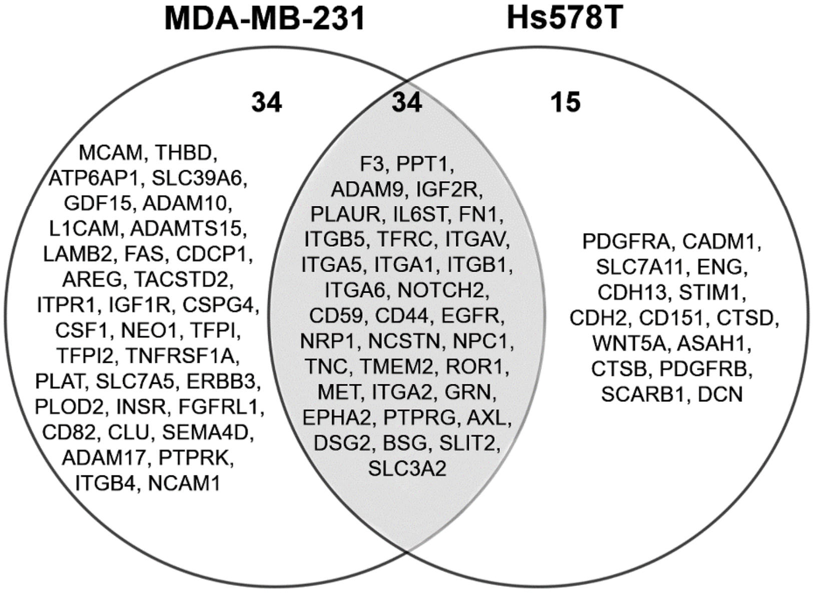

Cellular Growth and Proliferation

e

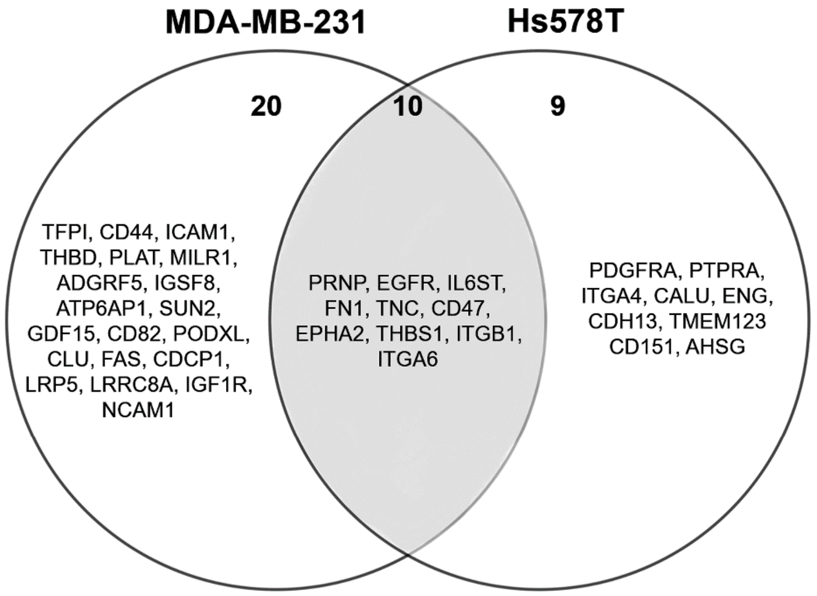

Cell Morphology

f

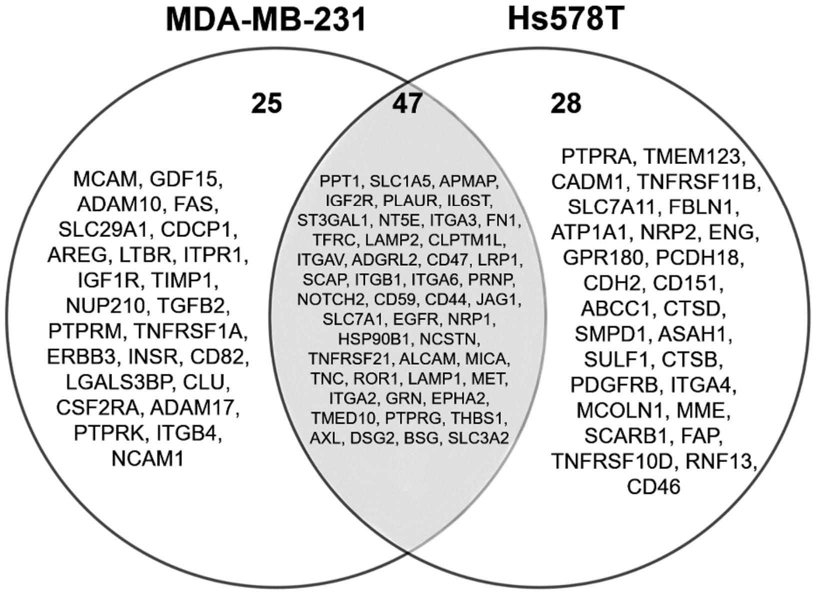

Cell Death and Survival

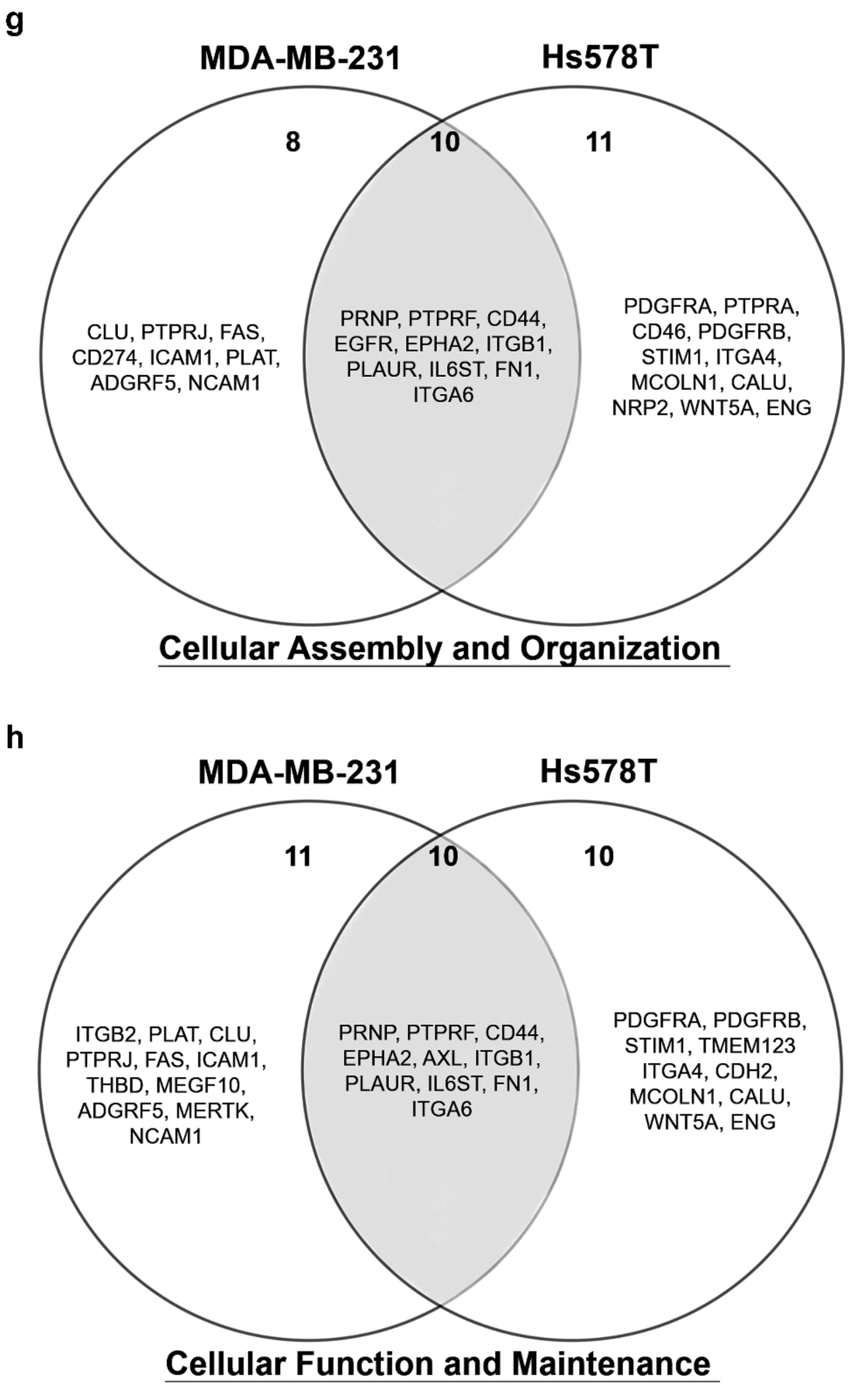
g
MDA-MB-231
Hs578T
8
10
11
CLU, PTPRJ, FAS, CD274, ICAM1, PLAT, ADGRF5, NCAM1
PRNP, PTPRF, CD44, EGFR, EPHA2, ITGB1, PLAUR, IL6ST, FN1, ITGA6
PDGFRA, PTPRA, CD46, PDGFRB, STIM1, ITGA4, MCOLN1, CALU, NRP2, WNT5A, ENG
Cellular Assembly and Organization
h
MDA-MB-231
Hs578T
11
10
10
ITGB2, PLAT, CLU, PTPRJ, FAS, ICAM1, THBD, MEGF10, ADGRF5, MERTK, NCAM1
PRNP, PTPRF, CD44, EPHA2, AXL, ITGB1, PLAUR, IL6ST, FN1, ITGA6
PDGFRA, PDGFRB, STIM1, TMEM123 ITGA4, CDH2, MCOLN1, CALU, WNT5A, ENG
Cellular Function and Maintenance

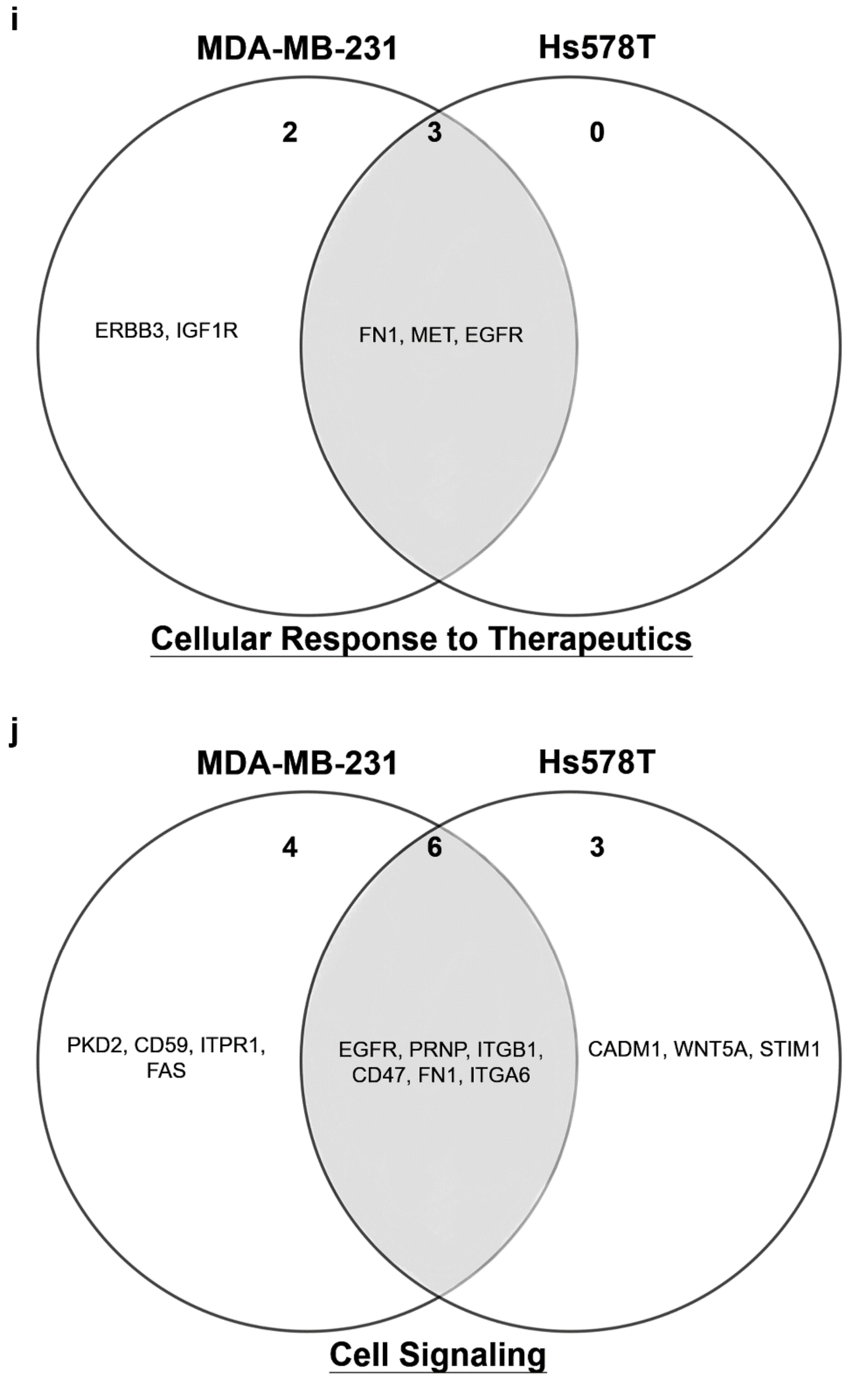

#

Figure S2. Venn diagrams show the FUT8 targets involved in the functions and identified in both invasive breast carcinoma cell lines. Grey regions show the intersection of the two sets.
