# Supplementary material for: Quantitative glycoproteomics analysis identifies novel FUT8 targets and signaling networks critical for breast cancer cell invasiveness
Source: Breast Cancer Res. 2022 Mar 18;24:21. doi: 10.1186/s13058-022-01513-3 (PMC8932202; doi:10.1186/s13058-022-01513-3)
Supplement: Supplementary file 5 — Additional file 5: Fig. S3. Validation of the SILAC results of identified glycoproteins. [file 13058_2022_1513_MOESM5_ESM.pdf]

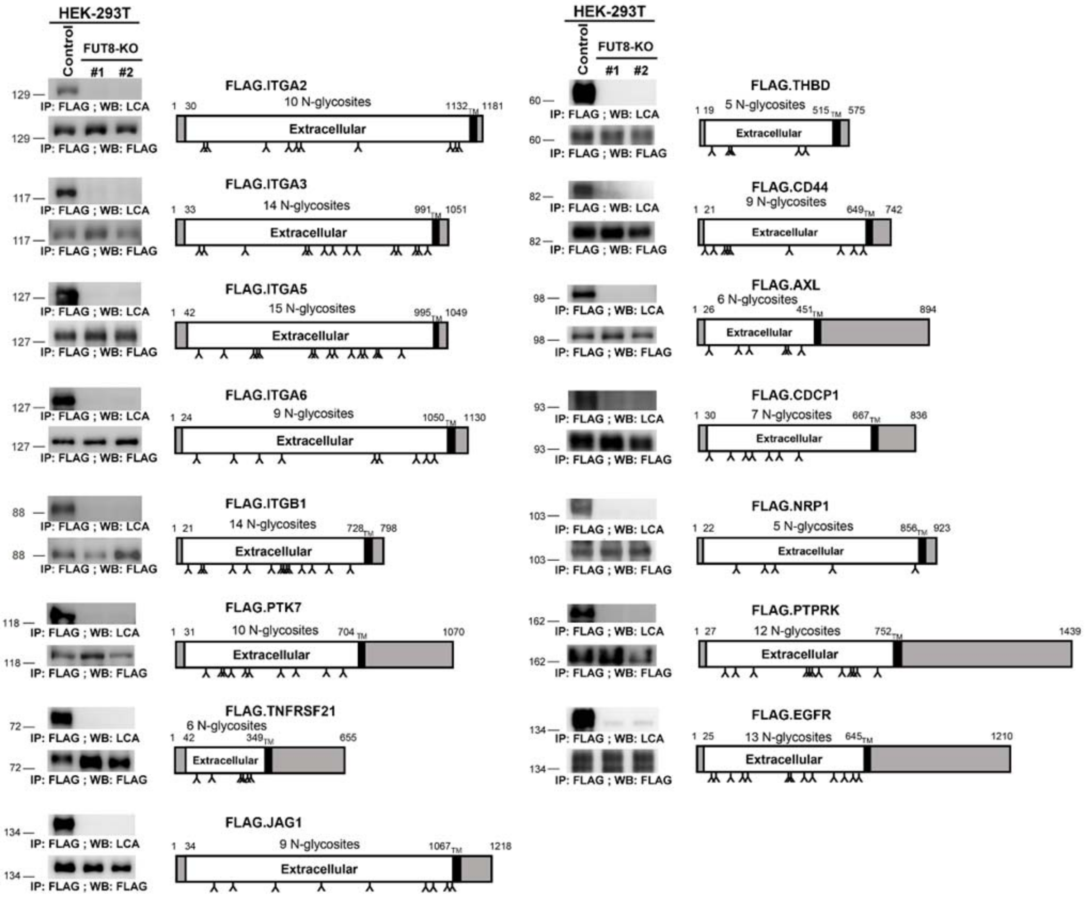

Figure S3. Validation of the SILAC results of identified glycoproteins. Core fucosylation of the selected proteins was eliminated by FUT8 knockout (KO). Recombinant proteins in the control or two FUT8-KO HEK-293T cell lines were probed with biotinylated LCA, then detected with streptavidin-conjugated horseradish peroxidase. Protein domain organization of these selected proteins was depicted according to the Uniprot database; potential N-linked glycosylation sites are marked. TM, transmembrane domain.
