# Supplementary material for: Quantitative glycoproteomics analysis identifies novel FUT8 targets and signaling networks critical for breast cancer cell invasiveness
Source: Breast Cancer Res. 2022 Mar 18;24:21. doi: 10.1186/s13058-022-01513-3 (PMC8932202; doi:10.1186/s13058-022-01513-3)
Supplement: Supplementary file 6 — Additional file 6: Fig. S4. Specific remove of core fucose but not terminal galactose in the FUT8-deficient cells. [file 13058_2022_1513_MOESM6_ESM.pdf]

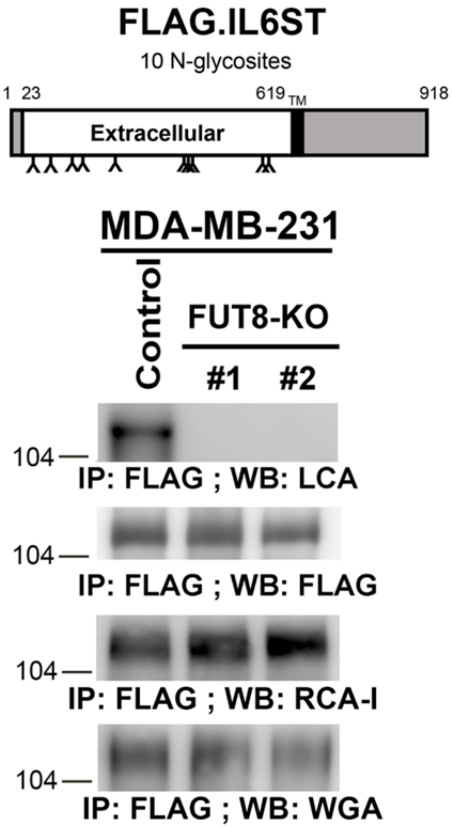

Figure S4. Specific remove of core fucose but not terminal galactose or N-acetylglucosamine in the FUT8-deficient cells. Recombinant FLAG-tagged IL6ST protein produced from control or two FUT8-KO MDA-MB-231 cell lines were probed with biotinylated LCA (for core fucosyaltion), RCA I (for terminal galactose), or WGA (for N-acetylglucosamine), then detected with streptavidin-conjugated horseradish peroxidase.
