# Supplementary material for: Quantitative glycoproteomics analysis identifies novel FUT8 targets and signaling networks critical for breast cancer cell invasiveness
Source: Breast Cancer Res. 2022 Mar 18;24:21. doi: 10.1186/s13058-022-01513-3 (PMC8932202; doi:10.1186/s13058-022-01513-3)
Supplement: Supplementary file 8 — Additional file 8: Fig. S5. Core fucosylation sites of integrin β5. [file 13058_2022_1513_MOESM8_ESM.pdf]

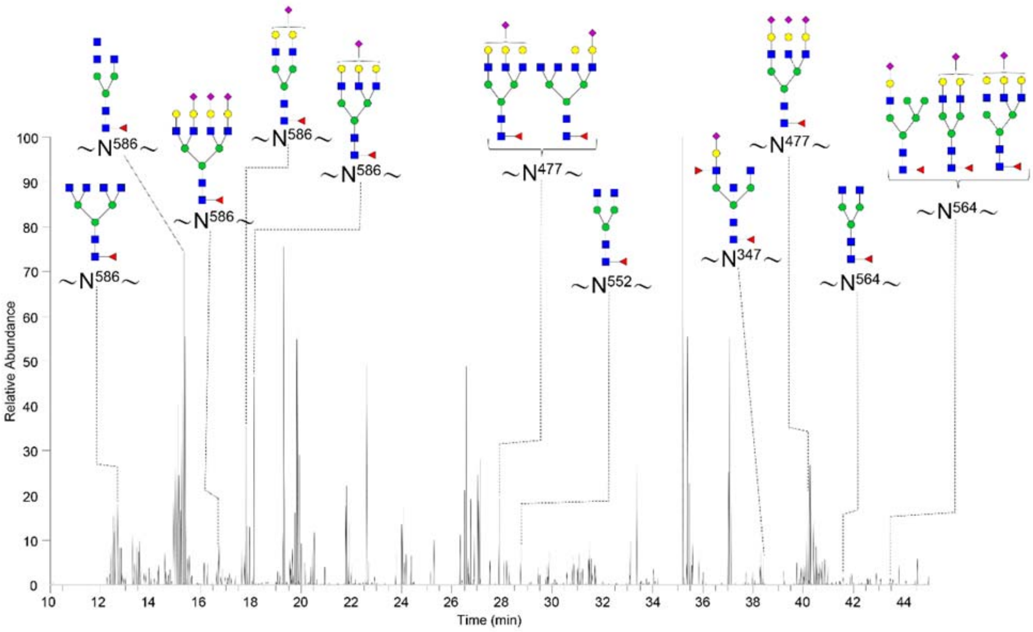

Figure S5. Core fucosylation sites of integrin β5. LC-MS/MS summed extracted ion chromatogram (XIC) of identified core-fucosylated glycopeptides for integrin β5. The number on asparagine (N) indicates the position in the protein sequence.
